# The Nutrition and Health Status of Residents of the Northern Regions of Russia: Outlook of Vertical Agricultural Farms

**DOI:** 10.3390/ijerph18020414

**Published:** 2021-01-07

**Authors:** Nikolay I. Didenko, Vladimir A. Davydenko, Elena R. Magaril, Gulnara F. Romashkina, Djamilia F. Skripnuk, Sergei V. Kulik

**Affiliations:** 1 Institute of Industrial Management, Economics and Trade, Peter the Great St. Petersburg Polytechnic University, 195251 St. Petersburg, Russia; didenko.nikolay@mail.ru; 2 Financial and Economic Institute, University of Tyumen, 625003 Tyumen, Russia; vlad_davidenko@mail.ru (V.A.D.); gr136@mail.ru (G.F.R.); 3 Department of Environmental Economic, Graduate School of Economics and Management, The Ural Federal University, 620002 Ekaterinburg, Russia; magaril67@mail.ru; 4 Humanitarian Institute, Peter the Great St. Petersburg Polytechnic University, 195251 St. Petersburg, Russia; kulik54@mail.ru

**Keywords:** vertical agricultural farm, food practices of the population in the northern regions of Russia, healthy food, environment

## Abstract

This paper is dedicated to studying the nutrition, health status and food provision of the people living the northern regions of Russia. The authors developed a concept of comprehensive interdisciplinary research of traditional and innovative behavioral practices of actors in the northern regions of Russia in the field of food production and consumption in order to study the structure of nutrition of the population, its health status and the technologies that are used to provide the people with food products. The interdisciplinary comprehensive research applied the following methods: (a) statistical method; (b) sociological method of mass survey; (c) sociological method of expert interviews; (d) method of feasibility study; (e) method of mathematical modeling. According to the results of the analysis, the nutrition of the people living in the norther regions is characterized by insufficient consumption of fresh vegetables, meat and processed meat, fish and seafood, milk and dairy products, some vitamins and bio-elements (such as selenium, calcium) and excessive consumption of saturated fats and flour products. The following problems related to providing the population of the northern regions of Russia with food products were identified: the agriculture in almost all northern regions of Russia has negative profitability; imported food products and food ingredients are mostly used; there are drawbacks of logistics, transportation and storage of food products; the natural and climate conditions are unfavorable for traditional agriculture. The paper substantiates the economic, environmental, social, and political advantages of highly automated agro-industrial complex of vertical farming as an alternative method for providing food security of the inhabitants of the northern regions of Russia.

## 1. Introduction

### 1.1. Why the Nutritional and Health Issues in Russia’s Northern Regions Should Be Investigated

The northern territories of Russia are an important source of natural resources whose intensive use results in a large influx of various groups of population that have to be provided with everything they need for life-sustaining activities. A most important condition for life-sustaining activities of humans in the Arctic is food security. Food security is understood both as the availability of sufficient nutrition and the presence of economically reasonable physical access to what is consumed. Food security is a situation when all people at all times have a physical, social and economic access to sufficient amounts of secure and high-quality food that satisfies their diet needs and food preferences for an active and healthy life [1]. Insufficient access to food causes health problems. If the lack of food pertains throughout a person’s entire life, it results in a deficit of cognitive abilities, reduction in physical labor productivity, illness at an older age, including chronic conditions, such as cardio-vascular diseases, diabetes and others. The quality of food, microelements needed by the human body is also very important ([2], p. 274). Poor quality of food or malnutrition are exceptional negative factors affecting human beings. In scientific literature they are defined as the absence of food security.

The change in climatic and sociocultural conditions has made local communities in the Arctic more dependent on the food products brought from other regions. It has had a significant negative effect on the population’s physical and mental health [3]. The extreme climate conditions have a massive impact on the people. Human bodies are forever suffering from oxygen deficiency, the effects of magnetic storms, the change of polar night and day, increased levels of radiation and strong electric fields. In comparison to the conditions of moderate climate, physical labor calls for higher energy costs. By and large, the main food products in the diet of the lower-income category of people are bread, cereals, and pasta, while fish and meat of marine mammals prevail in the villages of the northern coast of the Arctic.

The actual diet of the adult population in the northern regions, as has been proven by medical and sociological research, is not balanced. The actual diet combined with harsh weather and climate conditions and a difficult socioeconomic situation determines the specific features of the unfavorable and uncomfortable nutritional status of people in the northern regions. The actual diet lacks sufficient amounts of meat and meat products, fish and seafood, milk and dairy products, unsaturated fats, phospholipids, fiber, methionine, some vitamins and bio-elements (such as selenium, and calcium) while the consumption of saturated fats and flour products is abundant. The deficit is noted in the consumption of potatoes and other vegetables, fruit, and eggs [4,5,6,7].

The indigenous inhabitants of the Arctic consume larger quantities of bread, fatty foods, fish and lesser amounts of vegetables, milk and fermented milk products. They buy preserved diary and meat products more frequently than incomers. It is known that the biggest deficit from the recommended consumption rates is noted in relation to potatoes (48%), other vegetables and cucurbits crops (33%), fruit and berries (21%), eggs, milk and dairy products (about 14%), bread products (17%). It should be noted that the glucose loading of the inhabitants of the Arctic zone is high with sugar consumption exceeding the norm by 44% [8]. The high cost and a relatively bad quality of food available for remote northern communities have been a long-standing and acute problem of those living in the northern regions of different countries around the world [9].

Given that the health of the people living in the northern regions is directly related to the structure and quality of their nutrition, it is necessary to study the structure of the population’s nutrition, find out the link between the health and nutrition in the northern regions of Russia, and analyze how the food security provided to the population in the northern regions is organized.

### 1.2. Is There an Alternative to Traditional Agriculture in the Arctic?

Analyzing the chain “provision of the population of the northern regions with food products—the structure of nutrition of the population—the health status given the nutrition—the illness incidence dynamics”, we end up having to investigate the origins of food products in northern regions. Let us consider two areas: agriculture with traditional technologies and innovations of the 21st century—argoecological and other innovative approaches. Agriculture with traditional technologies is a basic sphere of human activities which provides population with food and gives jobs to a large proportion of people around the world. Lack of access to food provision can be solved using alternative methods, including the “agroecological” approach (“agroecological” approach [10], p. 203). The agroecological approach means searching for local solutions and rejecting stereotypes. Agroecological initiatives both have a regional character and a local one. Wild foods can also be a part of the structure of nutrition of human beings [11].

The specifics of food supply in the Arctic and the development of agriculture in the north of Russia include the limited availability of biological resources, unfavorable agricultural conditions (lack of warmth, short vegetation period, poor soils, excessive moisture). In the opinion of the leading experts of the *Institute of Socioeconomic and Energy Problems of the North, Komi*, the research center of the Ural Division of the Russian Academy of Science, this is something that prevents the population from being able to provide itself with local foods.

The food provision and development of agriculture in the north of Russia is supported from the federal budget. Ivanov et al. [12] suggest that the federal budget should support an increase in the quantity of cattle and deer; compensate, to some extent, the cost of modern machinery, high-performance equipment, mineral fertilizers, fuel, spare parts, feed compounds; the tariffs should be maintained at 50% during transportation of material and technical resources using railway and water transport; interest rates on loans should be subsidized; and subsidies should be granted to eliminate poverty among rural population.

The development of greenhouse farming is also supported. It has proven to be the most profitable area of agriculture in Russia over the last seven years. This business attracted investors’ attention after the devaluation of the ruble and food embargo in 2014. According to the Director of the National Union of Fruit and Vegetable Producers, the investments in greenhouses in 2015–2019 exceeded 200 billion rubles with more than 1100 ha of new greenhouses having been built [13]. Thus, to provide Chukotka with local foods, the owners of a greenhouse complex are constructing a new building. After it is finished, produce can be increased up to 27 tons a year. Eight tons of local vegetable produce was grown in 2019. If the price of cucumbers grown in greenhouses is approximately 480 rubles a kilo, the price of imported ones is 600 rubles a kilo. This price difference seems to be so impressive that the government believes: “It is 100% certain that vegetables should be grown locally” [14].

The second area is the innovations of the XXI^st^ century. Vertical farming has been discussed a lot in scientific literature recently. The theory and practice of growing crops in vertically stacked layers are also defined as a smart plant factory [15,16,17]. A major problem in the development of vertical farming today is studying and creating new biologically and economically efficient agricultural technologies [18,19,20,21]. The key criterion for the operation of a vertical farm is maximum efficiency with a minimum floor area [22,23]. That is the reason why using soil in vertical farms is unreasonable.

The modern concept of vertical farming was suggested by Despommier in 1999 [19]. Water solution containing fertilizers ensures growth of plants and is used instead of traditional soil. Various systems can be used to provide vertical farms with nutrients: hydroponic, aeroponic or aquaponic systems [24]. The systems do not contain soil. In hydroponic systems, the roots of plants are submerged in liquid solutions containing macroelements, such as: nitrogen, phosphorus, sulfur, potassium, calcium, magnesium, and microelements, including iron, chlorine, manganese, boron, zinc, copper, and molybdenum. An inert (chemically inactive) medium, like gravel, sand, sawdust is used as soil substitutes to hold roots [25]. The most effective system for growing plants on vertical farms is aeroponic, using 90% less water than the most effective hydroponic system. The US National Aeronautics and Space Administration (NASA) is in charge of this innovative indoor gardening technology. In the 1990s NASA was looking for effective ways of growing plants in space and invented the term aeroponics, defined as “the process of growing plants in an air or mist environment without the use of soil or an aggregate medium” [26]. Plants, grown in aeroponic systems, consume more minerals and vitamins, which makes them healthier and potentially more nutritious in comparison to other vertical farming schemes. An acquaponic version of vertical farms is obtained if plants and fish are united in a single ecosystem. Fish is grown in closed-circuit ponds, producing waste that is rich in nutrients, which is then used to feed plants on a vertical farm. Plants filter and treat discharge water, which is then returned to fish ponds. Fish provide food for plants and plants purify water. Aquaponics is used in small vertical farming systems with a majority of commercial vertical farming systems being focused on producing only a few fast-growing vegetable crops. This simplifies the problems of economy and production and maximizes efficiency. New acquaponic systems make this closed-circuit system more and more popular [27]. Hydroponic farms vary in their locations.

Vertical farms can have various shapes and sizes, from simple two-level to several-storey warehouses. They are often located in containers or abandoned buildings in cities, like, for instance, the farm of ILIOTEC company in Petropavlovsk-Kamchatsky (Russia). This farm was set up in the center of the city inside an old hangar, and its floor area is over 1600 m² [28]. In order to create vertical farms, new buildings are also erected. Farms, based on transportation containers, can deliver fresh produce all year round at a price lower than in case food is transported in the same containers from more southern regions. Many of such farms operate in the Arctic zones [29]. The common variants of structures for accommodating vertical farming systems also include tunnels and abandoned mine shafts. As was proven by Benke and Tompkins, the use of vertical farms together with other modern technologies helped to increase the yield in comparison with traditional agricultural methods by up to 50 times. The recuperation period of this new technology can be estimated as just 6–7 years [18]. In modern science, vertical farming is presented in the context of such key issues as the driving forces of innovation, potential and real advantages, controlled environment; biological, technical, technological and economic problems.

Vertical farms have amazing advantages: they are not dependent on the influence of weather conditions. Cereals can be grown in the most suitable environment round the year. Vertical farms do not require pesticides or herbicides, which means they produce no agricultural pollutants. With land agriculture being transformed into vertical farming, people do not have to worry about the lack of plough lands. Since farming is conducted in closed premises, people can grow harvest throughout a year without having to worry about bad weather, drought or natural disasters. From the perspective of food security, the advantages of vertical farming in comparison with traditional agriculture are obvious.

This paper investigates the connection between the structure of nutrition of the population and its health status in the northern regions of Russia, studies the options for organizing food security provision to the people living in the northern regions of Russia.

The research questions of the present study are stated below:

(1) Why nutritional and health issues in Russia’s northern regions should be investigated?
(2) Is there an alternative to traditional agriculture in the Arctic?
(3) How do residents of the region assess the quality of food?
(4) What are the links between food preferences and health?
(5) Are highly automated agro-industrial complexes of vertical farming a way to solve the problem of food security in the Arctic?

## 2. Materials and Methods

### 2.1. Research Methodology

The authors developed a concept of comprehensive interdisciplinary research of traditional and innovative behavioral practices of actors in the northern regions of Russia in the field of production and consumption of food products. The aim of the research was to study the structure of the population’s nutrition, the health status related to the nutrition and technologies providing food security.

The interdisciplinary comprehensive research applied the following methods: (a) statistical method; (b) sociological method of mass survey; (c) sociological method of expert interviews; (d) method of feasibility study; (e) method of mathematical modeling.

The sociological method of mass questionnaire survey (1610 people) was used to find out the concrete behavioral practices in the field of food consumption on the part of the inhabitants of a specific Arctic region, and to verify the relations between the self-assessments of health, behavioral practices and attributes of social status (health, labor and material status) at the micro-level.

The sociological method of expert interviews was carried out in the face-to-face format with the executives and employees of state and local government bodies, enterprises specialized in the making and processing of agricultural produce, and in retail selling of food products (50 people). The expert interviews were aimed at finding out the perceptions of the informed part of society about the main problems in the field of food consumption and food security, as well as evaluating its readiness for adopting and using innovative technologies in agriculture as a whole and for the method of vertical farms in particular.

The method of feasibility study is aimed at working out general technical and economic requirements for the stages of the life cycle of a highly-automated agro-industrial vertical farming complex in the northern conditions.

The northern conditions are as follows: harsh natural and climatic conditions, poor transport accessibility of the region, a low level of self-provision of food products and agricultural raw materials (crop farming products amount for less than 10%) with a high percentage of dissatisfaction with the supply of food products (about 71% in some districts), negative profitability of agricultural production, the industrial exploitation of the region leads to a reduction in territories suitable for traditional agriculture.

The method of mathematical modeling is aimed at giving a comprehensive assessment of vertical farming as one of the ways to solve the problem of food security in the northern regions of Russia. Vertical farming in the northern regions is considered in the model as a highly-automated sector which is intended to tackle the problems of food security in the northern regions and evaluate the economic, environmental, social and political advantages of vertical farming.

### 2.2. The Research Questionnaire 

The questions of the mass questionnaire used in the analysis are listed below. The percentage of the respondents is given in brackets, the “void” option is not stipulated.

(1) Note the settlement you live in.
(2) Your sex: 2.1. Male (44%) 2.2. Female (56%).
(3) Your age: |___________|
(4) How long have you lived in this city (town, village)?
  4.1. Up to 5 years (13%). 4.2. 5–15 years (17%). 4.3. 16–25 years (24%). 4.4. Over 25 years (46%).
(5) Please, assess the quality and safety of food products you buy in shops using a 5-score scale, where 1 is “very bad” and 5 is “excellent” $\underline{*}$:

5.1. Bread and flour products1 ÷ 55.9. Diary products1 ÷ 55.2. Pastry1 ÷ 55.10. Butter, cheese1 ÷ 55.3. Grocery products (flour, cereals, etc.)1 ÷ 55.11. Fruit and berries1 ÷ 55.4. Meat and processed meat products (apart from poultry)1 ÷ 55.12. Vegetables grown in a greenhouse1 ÷ 55.5. Poultry, processed products1 ÷ 55.13. Vegetables grown in open ground1 ÷ 55.6. Sausage products, smoked products1 ÷ 55.14. Alcoholic drinks1 ÷ 55.7. Fish and seafood1 ÷ 55.15. Soft drinks1 ÷ 55.8. Eggs1 ÷ 5

(6) Do you consume vegetables grown in open ground or in a greenhouse?
  6.1. I haven’t thought of it. I buy the ones available in shops and suitable in terms of price and quality (59%).
  6.2. It depends on the season: in the summer I buy vegetables grown in open ground, in the winter—in a greenhouse (34%).
  6.3. I only buy vegetables grown in open ground (2%).
  6.4. I only buy vegetables grown in a greenhouse (2%).
(7) Do you grow your own vegetables for personal consumption? 7.1. Yes (14%). 7.2. No (86%).
(8) Note your preferences when you buy food products. Choose one of two options in each line (the “void” option is not stipulated):

*Do you prefer food that is:*

8.1.1. Packaged (76%)8.1.2. Non-packaged (20%)8.2.1. Non-processed (48%)8.2.2. Primarily processed (38%)8.3.1. Preserved (26%)8.3.2. Containing no preservatives (70%)8.4.1. Raw foods (60%)8.4.2. Semi-finished foods (25%)

(9) Which of the listed food products have you and your family members bought over the last 7 days?

9.1. Bread and flour products92%9.10. Butter, cheese44%9.2. Pastry48%9.11. Fruit and berries48%9.3. Grocery products (flour, cereals, etc.)52%9.12. Vegetables grown in a greenhouse51%9.4. Meat and processed meat products (apart from poultry)25%9.13. Vegetables, grown in open ground30%9.5. Poultry, processed products59%9.14. Alcoholic drinks21%9.6. Sausage products, smoked products54%9.15. Soft drinks41%9.7. Fish and seafood22%9.16. Canned foods and semi-finished foods (not including meat and fish tins)11%9.8. Eggs42%9.17. Oil34%9.9. Diary products76%9.18. Drinking water32%

(10) Which measures do you think are mostly needed to provide food security in the northern regions (choose no more than three most important measures in your opinion):
  10.1. Supporting the food producers for the northern regions on the part of the government (57%).
  10.2. Stimulating the inhabitants of the northern regions to grow their own foods (29%).
  10.3. Supporting the technologies of the 21st century to develop agriculture in the North (25%).
  10.4. Developing traditional greenhouse farming with artificial lighting and heating in the North (29%).
  10.5. Developing vertical farming as a method when fertilized water solution is used instead of traditional soil and thanks to that plants are grown in a small space (5%).
  10.6. Developing logistic networks and trade in fresh and high-quality food products (42%).
  10.7. Providing the low-income population with material support to buy high-quality food products (76%).
  10.8. Development of the traditional economy of the North peoples (11%).

(11) Which of the following statements best characterizes your financial situation today—yours, your family?
  11.1. Money is tight for everyday expenditure (6%).
  11.2. All the salary is spent to cover everyday costs (14%).
  11.3. Money is enough for everyday costs, but buying clothes is difficult (19%).
  11.4. Money is mostly sufficient, but we have to borrow to buy expensive items (25%).
  11.5. Money is enough for virtually everything, but buying an apartment or a country house is difficult (25%).
  11.6. We can afford virtually anything (9%).

(12) Your marital status?
  12.1. Married (55%).
  12.2. Divorced (11%).
  12.3. Living together but the marriage is not registered (10%).
  12.4. Single (17%).
  12.5. Widower/widow (7%).

(13) How many children do you support?
  13.1. None (49%).
  13.2. One child (23%).
  13.3. Two children (17%).
  13.4. Three or more children (10%).

(14) Your education:
  14.1. Incomplete secondary (5%).
  14.2. General secondary (12%).
  14.3. Vocational secondary (40%).
  14.4. Higher and post-graduate (43%).

(15) How do you assess your health status?
  15.1. Very bad (14%).
  15.2. Rather bad (40%).
  15.3. Quite good (29%).
  15.4. Apparently healthy (17%).

The questions of expert interviews used in the analysis of this survey (50 interviews):

(1) How do you assess the possibilities for the development and the efficiency of agriculture in the northern regions?
(2) Can the northern regions fully or partially satisfy its needs for fresh vegetables? Other food products?
(3) Are you aware of innovative facilities for developing the agriculture in the northern regions?
(4) Do you know what vertical farms are and how do you assess the potential of applying them in the northern regions?

### 2.3. Analysis of Primary Information

The sample contained the population of the northern region aged over 18. 1610 people were interviewed, with 44% of them being males and 56% females, and 43% had higher or advanced tertiary education. The sample error by one feature Δ = 2.8%. The statistical analysis was performed using SPSS package, Statistics version 25 (IBM Corporation, Armonk, NY, USA); value *p* < 0.05 was considered statistically significant. The methods of statistical analysis used the factor assessments of the respondents concerning the quality of food products the local residents were buying in the nearby shops. The key criterion was the level of food sensations. The normality of feature distribution was checked by the Mann-Whitney U-test. The Fisher angular transformation was used to evaluate the significance of differences between the sample rates by one feature. The critical significance level p in all the procedures of statistical analysis was taken equal to 0.05 (Fisher’s test = 12.38, Pearson’s chi-squared test = 0.34). The authors used standard theoretical sources and practical applications to tackle the problem of data analysis, relying on the world experience in similar sociological and statistical studies [30,31,32,33].

## 3. Results

### 3.1. The Quality of Food Products According to the Assessments of the People Living in the Region

**Figure 1:**
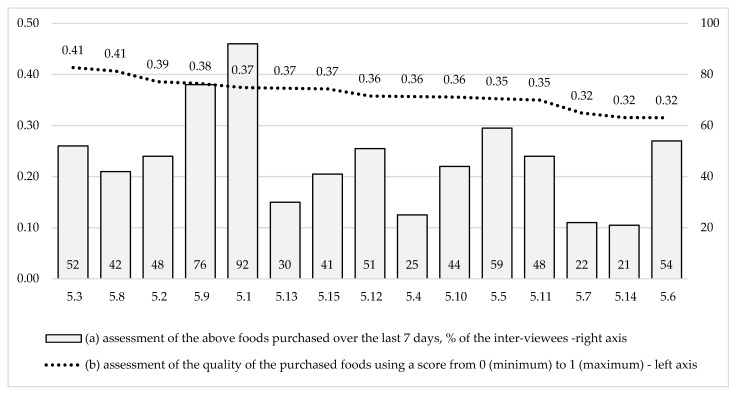
Assessing the quality of food products: (**a**) assessment of the above foods purchased over the last 7 days, % of the interviewees; (**b**) assessment of the quality of the purchased foods using a score from 0 (minimum) to 1 (maximum). Source: according to the data of the authors’ sociological survey. The scale was transferred into the interval 0 (very bad)—1 (excellent). 5.1. Bread and flour products. 5.2. Pastry. 5.3. Grocery products (flour, cereals, etc.). 5.4. Meat and processed meat products (apart from poultry). 5.5. Poultry, processed products. 5.6. Sausage products, smoked products. 5.7. Fish and seafood. 5.8. Eggs. 5.9. Diary products. 5.10. Butter, cheese. 5.11. Fruit and berries. 5.12. Vegetables grown in a greenhouse. 5.13. Vegetables grown in open ground. 5.14. Alcoholic drinks. 5.15. Soft drinks.

The assessment of the food quality by the residents of the region includes the following research questions: (a) How do the inhabitants of the northern region evaluate the quality of foods they buy in shops? (b) How are the assessments of the food quality related to the shopping frequency? (c) How are the assessments of the food quality related to the major socio-demographic characteristics? (d) Which characteristics are the most important for the consumers when they choose high-quality food? Figure 1 shows the assessments of the shopping frequency and quality of food products.

It is important to understand that agriculture has negative profitability virtually in all the northern regions of Russia. According to the data of the Single Statistics Database in 2017 the profitability of agriculture was minus 60% in Yamal-Nenets Autonomous District, minus 30% in the Republic of Sakha (Yakutia), minus 52% in Chukotka Autonomous District, and minus 18% in Nenets Autonomous District. Thus, the people are mostly provided with imported foods.

The basis of the food basket (the first group of food products) of the residents of the northern region (over 50% of weekly purchases) is bread (92%), milk and fermented milk products (76%), poultry and processed poultry products (59%), dried products and pasta (52%), sausage and smoked products (54%), vegetables grown in a greenhouse (51%). From 30 to 49% of weekly purchases (the second group of food products) include confectionery, fresh fruit and berries, cereals (48%), butter and cheese (44%), eggs (42%), soft drinks (41%), oils (34%), vegetables grown in open ground (30%). The most infrequently purchased foods are meat and meat products (25%), fish and seafood (22%), alcoholic beverages (21%), tinned and prepared food (11% and less), Figure 1, the right axis.

The basis of the food basket (over 50% of weekly purchases) is virtually the same for three top income quartiles. The lowest income quartile demonstrates a growth in the consumption of bread to the disadvantage of all other types of food: bread (96%), milk and fermented milk products (68%), poultry and processed poultry products (51%), dried foods and pasta (50%). The second group comprises cereals (48%), sausage and smoked products (42%), vegetables grown in a greenhouse (34%), and oil (34%). All other products are in the category of rarely purchased. The consumption of pasta and bread is approximately the same in the five bottom segments and goes down a lot in the top segment. It can be concluded that the reduced consumption of pasta and bread in our society differentiates the wealthiest ones. More frequent purchases of meat and meat products (10% above the average) is distinctive in the two top material and property segments.

The structure of the food basket of the youngest demonstrates a gradual shift in priority from bread, cereals (−5%) and pasta to milk and fermented milk products (+8%), chicken or other poultry (+6%), fresh tomatoes, cucumbers, fruit and berries (+5%), drinks of all kinds. The frequency at which drinks are bought, both alcoholic and soft, reduces as the age of the respondents goes up. For example, the frequency of buying soft drinks is 55% and 45% in the groups aged 18–24 and 25–44, respectively, and 29% in the group aged 55+. As for the segment of alcoholic beverages, it is 25% in the group ages 18–34, and 15% in the group aged 55+.

The assessment of the quality and safety of food products by the inhabitants of the northern territories does not coincide with the structure of the food basket. The average score of the quality and safety of food products is equal to 0.36 on scale from 0 (minimum) to 1 (maximum trust in quality and safety), Figure 1, left axis. The leaders in the quality assessment scores are dried foods, cereals and eggs (0.41), confectionery and dairy products (0.39–0.38), bread and flour products, vegetables and soft drinks (0.37). The minimum quality assessment scores were given to sausage and smoked products, fish and seafood, alcoholic drinks (0.32). The consumption of fish and seafood is in the tail of needs and the people cut down on these foods most of all.

The assessments of the quality and safety of food products are highly suitable for calculating mean values. Cronbach’s alpha coefficient is equal to 0.93 by 15 items. The type of settlement, financial situation and age have the greatest influence on the average scores. The lowest value of the scores assessing the quality of food is given by the residents of rural areas (0.29), the population aged over 55 (0.34), and the poorest (0.32).

The requirements the consumers set down to the level of processing of food products are as follows. 70% select preservative-free food products; 60% prefer raw foods to those containing preservatives; 76% prefer packaged foods. As for processing, the choice of foods is less defined: 48% prefer non-processed and 38% processed foods. What is more, 15% of the respondents could not choose between raw food and semi-finished products, and 14% failed to choose between processed and non-processed foods.

The self-provision of the inhabitants of the northern regions with food products is at a low level. According to the sociological surveys, the problem of food quality annually goes in the top five problems experienced by the people living in rural regions. According to the authors’ survey, in 2019, 71% of the respondents highlighted the problem of the lack of high-quality food products in villages. The rural areas do not have enough high-quality and fresh food products, primarily, dairy products, fruit, and vegetables during the transitional fall and spring period (October–December and April–June), as well as in the period of summer vacations (July–August), which, in fact, amounts to 2/3 of a year. An additional constraint preventing the people from producing their own food products is the low level of readiness of the population to take part in agricultural production not belonging to the traditional sectors of agriculture. Less than 1% of the population living in the northern regions is ready to participate in dairy and vegetable agricultural production.

### 3.2. Analyzing the Relationship between Food Preferences and Health Status

Let us look into how food preferences affect the self-assessments of the consumers’ health status (Table 1).

Obviously, the lower the self-assessments of health status, the lower are the assessments of the quality and safety of the food products people buy in shops, Table 1. The dispersion analysis and Pearson’s chi-squared test confirm the statistical stability of the results. A more detailed analysis of the sociological survey data shows that ill people buy fresh fruit and vegetables, berries, dairy products and meat much more seldom than the people who find themselves apparently healthy. At the same time, the self-assessments of health status are not related to purchasing such products as dried food, bread, sausage products, alcoholic or soft drinks. The latter can be explained by the fact that the income of the people who often fall ill is lower, as a rule. The consumption of foods from the second group is pretty much independent on the level of income. Elastic by income, self-assessments of the health status and education is the consumption from the first group—fresh fruit and vegetables, berries, dairy products and meat.

**Figure 2:**
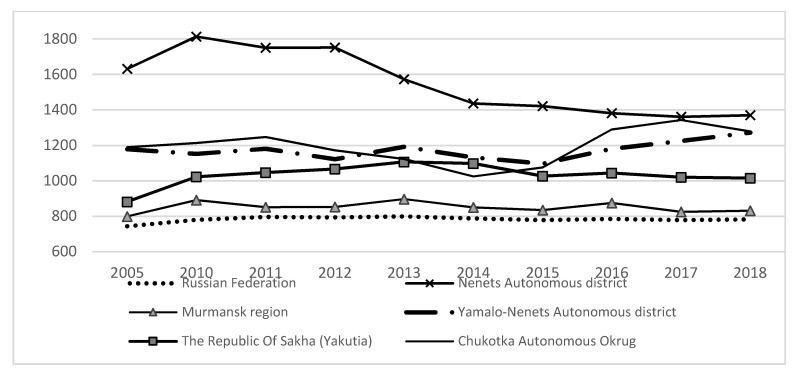
Incidence rate per 1000 population (registered illnesses of patients with a diagnosis established for the first time in life). Source: Russia’s regions. Socioeconomic indicators. Statistics digest/Rosstat—M., 2019.—1204 p. PP.391–394.

The illness incidence dynamics per 1000 Russia’s total population and the population in the regions of the Russian Arctic is presented in Figure 2. We should note that the diet of the inhabitants of Murmansk Region and Yakutia is far more balanced than that of the people living in other Arctic regions. They get sick much less often and the infant mortality is lower in these regions. Nenets Autonomous District (NAD) stands out visibly with the illness incidence rate being more than twice of the average in Russia until 2010. Then the incidence rate reduced but still remained at a very high level, exceeding the average Russian rate by 1.8 times.

Thus, the authors’ sociological survey on the actual nutrition of the inhabitants of the Arctic region, which was conducted using the method where the food consumption frequency was analyzed, confirms the conclusions made earlier in other empirical research studies, namely that the actual diet of the adult population in such an Arctic region as Yamalo-Nenets Autonomous District is unbalanced and lacks fresh vegetables, meat and meat products, fish and seafood, milk and dairy products, and unsaturated fats. The people consume too much oils and fats, bread products and sterols given a relatively stable quality of food products.

These conclusions are proven based on the analysis of the expert interviews. All the experts highlighted the growing importance of the health and demographic problems of the population living in the Russian Arctic. The experts (over 80%) point out that the main problem of food security is the unbalanced and low-quality nutrition of the population. The relation between the health of the population and behavioral practices towards food products is confirmed by us at the meso-level (that of the region) and the micro-level (self-presentation of the population).

### 3.3. Responses in the Expert Interviews Included in the Analysis, 50 Interviews

Typical responses of the 50 expert interviews are given.

#### 3.3.1. How Do You Assess the Possibilities for the Development and the Efficiency of Agriculture in the Northern Regions?

*Farming on Yamal is quite risky, because very different force-majeure situations are possible. If somewhere else they may be less noticeable, here they are sensed, of course. We have a complicated transportation scheme and 10 months of winter. I believe you have to be patient, inquisitive and optimistic to do farming on Yamal.*

*The main problems include the inaccessibility, remoteness and lack of access to cheap resources, starting from energy resources and labor resources. High obligations of the employer, namely the need to comply with the state tasks (increased wages in the northern regions, providing additional perks).*

*The incomes of enterprises in the Arctic zone are practically the same as those of the similar enterprises operating in central Russia.*

*Our enterprises have to spend more on energy resources.*

*As for agriculture, it cannot exist without state support.*

*A small navigation period limited by the local consumer does not allow the market to seriously grow.*

*No agriculture is possible in the North without state support.*

*No doubt, there are small farms, personal farms (not so many), which operate independently on a small scale and live without state support. Many of us grow quails and chickens.*

*The industrial exploitation of the North has an impact, of course, but this impact is not extremely significant, because we have checked and the lands allocated do not take up more than 7% of deer pastures. The degradation of the pastures is caused by large herds of deer, because the needs of the population are growing, the same as the number of families. Every family need their own herd, they are forever increasing the number of deer, but the resources are finite, the deer trample more than they eat. This problem has not arisen now, of course. The lack of pastures was discussed already 30 years ago.*

*The experience of the large enterprise of agro-industrial complex in our territory was not successful. In our territory, the smaller the size of an enterprise, the better it will function. This is the main distinction between the northern agricultural enterprises from those on the mainland.*

*I have a feeling that the rural territories are deeply stagnated, they do not develop or degrade. I. e. all the efforts make it possible to constraint the population while its growth is mainly determined by the birth rate among the indigenous low-numbered peoples of the North. Their birth rate indicators are a lot higher than the average ones.*

#### 3.3.2. Can the Northern Regions Fully or Partially Satisfy Its Needs for Fresh Vegetables? Other Food Products?

*No, the local population cannot provide itself with fresh vegetables, neither partially nor fully.*

*The crop-growing sector is practically unimportant for us, even though we produce somewhat 800 tons of potatoes may be in a couple of municipalities and mostly by the households of the population in the southern part of our region.*

*It seems that in case of cattle-breeding, we import everything: fodder, hay, feed compound. It is the north and nothing grows here well. And in most cases it is being developed for children’s dairy kitchens, for boarding schools, kindergartens. We also have fur-farming, but due to the social character there are just 2 or 3 farms left. The biggest industries are deer breeding and fishing.*

*I think that today the population of Yamal has access to, probably, everything, because the trade industry is very well developed. One of the main lines of business is providing people with food products. Of course, when it comes to crop-growing, the locally grown potatoes account for just 0.4% of the total need of the population, everything else is imported. The same is true with regard to milk. The biggest percentage falls to the share of fish products with about 70% of the population’s need being met with our own raw products. As for meat, we only have deer, everything else is imported. I wouldn’t judge about its quality. There are high-quality and poor-quality products.*

#### 3.3.3. Are You Aware of Innovative Facilities for Developing the Agriculture in the Northern Regions?

*In our land digitalization is just about electronic tags for deer.*

*Today innovative technologies are livestock complexes. Muck is disposed using a bioreactor, after which it can either be used as fertilizer or a secondary raw material.*

*The only thing I am aware of in terms of innovations is electro-shepherds.*

*We are forever investigating and inventing things. For example, my enterprise has a dryer working on dead waste, i.e., grain is dried, the remainder is straw, which is burned in the furnace and direct furnace gases dry the grain. There are no analogues of this machine in the world. It was customized to meet our requirements. I.e. the furnace is heated and the furnace gases first heat water and then the water heats the heating system.*

#### 3.3.4. Do You Know What Vertical Farms Are and How Do You Assess the Potential of Applying Them in the Northern Regions?

*No, we do not know or heard of them (55%).*

*Yes, we have heard of them, but we don’t know how they are used (20%).*

*If we have these technologies, we have some interesting capacities for using them. First of all, we have gas. Second, there is need for developing technologies with economical use of ground. Third, there are social problems, like employment of the local population. We can’t keep on growing the number of deer, because the pastures have already been running out of turnover due to overexploitation (15%).*

*There is need for providing local schools and kindergartens with fresh vegetables. The navigation is extremely limited in time. Developing such technologies would be very promising (10%).*

## 4. Discussion

### 4.1. The Concept of Comprehensive Interdisciplinary Research of the Structure of Nutrition, Health Status and Food Provision of the Population in the Northern Regions of Russia

Based on the developed concept the authors analyzed the chain “current food provision of the population in the northern regions—the structure of the nutrition of the population—the health status given the nutrition—illness incidence dynamics—solving the problem of food provision of the population in the northern regions of Russia.

The interdisciplinary comprehensive research applied the following methods: (a) statistical method; (b) sociological method of mass survey; (c) sociological method of expert interviews; d) method of feasibility study; (e) method of mathematical modeling.

As a result of the statistical, sociological method of mass survey, sociological method of expert interviews used in the process of research, the answers to the following questions were obtained: (a) How does the population of the region assess the quality of food products? (b) What is the connection between the food preferences of the population in the north region and its health? (c) What is the attitude of the inhabitants of the region towards highly-automated agro-industrial complexes of vertical farming as a technology capable of solving the problem of food security in the Arctic.

The method of feasibility study is aimed to analyze the technical and economic requirements for the stages of the life cycle of a highly-automated agro-industrial vertical farm in the conditions of the North. The technical and economic requirements for the stages of the life cycle as follows: extreme natural and climatic conditions of the North; the air temperature in the winter is from −20 °C to −40 °C; roads are covered in snow; maintenance facilities and safety; security threat of the population; the availability of the heat, gas and water supply services; the possibility of dangerous natural disasters like earthquakes, which cause partial or complete destruction of objects.

Vertical farming has been demonstrated in experimental scale and on the production level. It has potential advantages over agriculture. including the use of hydroponics, which places in question the need for soil farming of quite a few crops. This method of farming results in higher yield, which depends on the quantity of layers in a high-rise building or the number of stacks in a high-rise enclosure. Many of the auxiliary technologies were studied in former variations of greenhouse facilities, but today technologies are united in commercially viable systems due to rapid recent advances in electronics, mechanical engineering, solar and wind energy, batteries, light-emitting diodes, water recirculation and computing capacities.

The method of mathematical modeling is aimed at giving a comprehensive assessment of vertical farming as one of the ways to solve the problem of food security in the scale of all northern regions of Russia. This paper only contains the formulation of the model.

### 4.2. How the Population of the Region Assesses the Quality of Food Products 

The population is mostly provided with imported food products because the agriculture almost in all northern regions of Russia has negative profitability. The respondents attribute three components to the group of the most important characteristics of food products: freshness and shelf life of the product (81%), price (79%) and taste (60%). The place of production is important for 37% of the respondents, the presence of genetically modified foods for 33%, the presence of preservatives for 29%, the packaging, trademark and brand of the producer are important for 17% of the respondents. Freshness and shelf life are virtually the same important for all social groups. The price, which is expected, is less significant for those categories of the population that have a comfortable income. The price is a critical characteristic in the choice of food products for the people who live in small towns of the region, for the elderly (respondents aged over 65), for young people aged less than 24, and for the poor strata of the population. However, as the material status improves, the taste qualities of food products are getting more and more significant for the consumers. Thus, in the poorest group, the taste qualities of food are considered by 47% of respondents, while in the group of the wealthiest this number amounts for as many as 75%. The trademark or brand as a factor affecting the choice of food products, on average, has the least effect on the selection of food products. The trademark or brand is considered by just 17% of consumers, but among the wealthiest, as many as 26% take into account this characteristic when buying food. Moreover, the youngest respondents (31%) pay attention to the trademark or brand of the product. For the middle-aged customers, the place of production and eco-friendliness of products are more important with 20% assessing their importance in the age category of 18–24, 26% of the respondents aged 25–34 and 35% aged 35–44, 33% aged 45–54 and 28% aged over 55.

### 4.3. What Is the Connection between the Food Preferences and the Health of the Population in the Northern Regions

The health of the inhabitants of the northern regions is directly dependent on the structure and quality of their nutrition. The basis of the food basket of the population in the northern regions is bread, milk and fermented milk products, poultry and processed poultry, grocery products and pasta, sausage and smoked products, and vegetables grown in a greenhouse. The basis of the food basket is virtually the same for the three upper income quartiles. The lower income quartile demonstrates growing consumption of bread to the disadvantage of all other types of food products. It is highlighted that in comparison with the older generation, the younger consume almost twice as many vegetables and fruit, which are so important in the structure of nutrition.

The worsening of the health status of the population in the North, including due to the natural and climatic factors. Over the last decade, the incidence rate has been growing by all main classes of disease, primarily diseases of blood circulation organs, cardio-vascular and gastrointestinal diseases. Acute heart attack accounts for most illness incidence and deaths in the North 15 years younger than in central Russia. Due to the ozone layer disappearance, the doses of ultraviolet radiation rise and cause more cases of cataract and increased risk of skin cancer. Providing urgent medical care is difficult in the majority of the remote northern regions because sanitary aviation almost stops operating.

### 4.4. What Is the Attitude of the Population of the Region to Highly-Automated Agro-Industrial Complexes of Vertical Farming as a Technology Capable of Solving the Problem of Food Security in the Northern Regions

Let us highlight some problems:

Low awareness of the society. When answering the questions, only 5% of the inhabitants of the region showed any knowledge of this method. The informed part of the society includes experts, and government or economic structures. 55% heard of vertical farming, 20% heard something but do not know anything concrete. At the same time the potential of vertical farming is assessed quite highly. The experts noted: “If such technologies exist, we have some interesting capacities for using them. First of all, we have gas. Second, there is need for developing technologies with economical use of ground. Third, there are social problems, like employment of the local population. We can’t keep on growing the number of deer, because the pastures have already been running out of turnover due to overexploitation”. According to the local government, the northern regions “need to provide local schools and kindergartens with fresh vegetables. Our navigation is extremely limited in time. Developing such technologies would be very promising.” The low transport accessibility of the region, the low level of self-provision with food products and agricultural raw ingredients (the crop-growing produce is less than 10%) and high dissatisfaction with the supplied food products (up to 71% in some regions), the negative profitability of agricultural production, and the industrial exploitation of the region contribute to the reduction in the territories suitable for traditional agriculture.

In the experts’ opinion, the local population cannot provide itself with fresh vegetables neither partially, nor fully. “The crop-growing sector is practically unimportant for us, even though we produce somewhat 800 tons of potatoes may be in a couple of municipalities and mostly by the households of the population in the southern part of our region.” “It seems that in case of cattle-breeding, we import everything: fodder, hay, feed compound. It is the north and nothing grows here well. And in most cases it is being developed for children’s dairy kitchens, for boarding schools, kindergartens. We also have fur-farming, but due to the social character there are just 2 or 3 farms left. The biggest industries are deer breeding and fishing.” “I think that today the population of Yamal has access to, probably, everything, because the trade industry is very well developed. One of the main lines of business is providing people with food products. Of course, when it comes to crop-growing, the locally grown potatoes account for just 0.4% of the total need of the population, everything else is imported. The same is true with regard to milk. The biggest percentage falls to the share of fish products with about 70% of the population’s need being met with our own raw products.”

Weak innovativeness of the society. When answering the questions about which innovative technologies applicable in the agriculture of the northern regions they know or have ever heard of, the respondents gave very limited answers. “In our land digitalization is just about electronic tags for deer.” “Today innovative technologies are livestock complexes. Muck is disposed using a bioreactor, after which it can either be used as fertilizer or a secondary raw material.” “The only thing I am aware of in terms of innovations is electro-shepherds.” “We are forever investigating and inventing things. For example, my enterprise has a dryer working on dead waste, i.e., grain is dried, the remainder is straw, which is burned in the furnace and direct furnace gases dry the grain. There are no analogues of this machine in the world. It was customized to meet our requirements…”.

The people living in the northern region are not ready to use vertical farming because they are totally unaware of its capabilities. However, there is a large potential of its application. First, people prefer raw fresh food the safety and quality of which they can be sure of. Second, there is a large potential for developing food self-provision in the region. Third, over 60% of the experts highly value the potential of vertical farming in the northern regions. The experts believe that the population and business must be informed about its capabilities (51%), state support should be given to those who apply it (42%), and special infrastructure must be developed (16%).

Despommier pointed out a number of reasons why vertical farming could be very attractive for politicians [19,20,21]. Moreover, absolutely fantastic horizons were opening up in the context of space exploration [34] and from the perspective of economic, environmental, social and political aspects [23,35,36]. The arguments put forward by Cox, [37] have become less relevant because of the continuous progress in technology. New LED sources considerably increase yield [38]. The number of years since launch, i.e., the break- even point can be estimated as 6–7 years. [18] (p. 21).

### 4.5. Further Research

Let us return to discussing the advantages of the concept of a highly-automated agro-industrial complex (vertical farming) as an alternative method of food security. Despommier’s original vision of the advantages of vertical farming was based on the idea that it should be a world full of skyscrapers with several levels where crops are grown continuously round the year. The claimed advantages of vertical farming can be categorized and generalized from the perspective of economic, environmental, social and political aspects [23,35,36] and presented in a model.

Economic benefit. Vertical farming can give a competitive advantage as it combines extensive research and development with the experience in agriculture, big data and modern technologies increasing productivity.

*The environmental advantages* are considerable, including provision of healthy organic food, not contaminated by chemicals.

*The social advantages* are numerous too, because vertical farming provides new jobs in the field of biochemistry, biotechnology, mechanical engineering, agriculture, civil engineering, technical maintenance, and new opportunities for research and development aimed at improving the technology.

*The political advantages* of vertical farms are that the obligations to fight climate change will be easier to comply with, while new technologies will help the adaptation and mitigate harmful effects.

The main conclusions and generalizations concerning vertical farming make it possible to formulate an approach for a comprehensive assessment of vertical farming as an alternative method of food security in the Arctic. The comprehensive assessment of vertical farming is carried out using a model of interrelated econometric equations. The model is formed in case of the following prerequisites:

(a) The process of vertical farming is assessed using a system of indicators—a system of endogenous variables (denoted as $y_{t}^{k}$);
(b) The prehistory of the process exists, i.e., each endogenous variable is affected by the values of the endogenous variable in the preceding periods (denoted as Y (j-t));
(c) Endogenous variables have mutual influence on each other;
(d) Each endogenous variable is affected by internal and external factors assessed by exogenous variables (denoted as x_i_).

Endogenous (dependent) variables are the values of the variables that are determined inside the model or interdependent (y) [39]. Exogenous (independent) variables are the values of the variables that are set “externally”, autonomously. To a degree, they are controlled (planned) (x). It is essential that the dependence of endogenous (dependent) variables on exogenous (independent) variables should be established. In order to do so, certain endogenous variables are chosen and an assumption is made about their dependence on certain exogenous variables. Thus, after the analysis is carried out and the prerequisites are formulated, a dynamic model, describing a change in endogenous and exogenous variables, can be presented. In our analysis, the following ones are chosen as endogenous variables:

$y_{t}^{1}$ is the economic advantages of vertical farming of a system in the *t*-th year;
$y_{t}^{2}$ is the environmental advantages of vertical farming of a system in the *t*-th year;
$y_{t}^{3}$ is the social advantages of vertical farming of a system in the *t*-th year;
$y_{t}^{4}$ is the political advantages of vertical faming of a system in the *t*-th year.

Exogenous variables are as follows:

$x_{t-i}^{1}$ is the consumption of water of a system in the *t-i*-th year;
$x_{t-i}^{2}$ is the electricity costs of a system in the *t-i*-th year;
$x_{t-i}^{3}$ is the logistic costs of a system in the *t-i*-th year;
$x_{t-i}^{4}$ is the costs of land lease of a system for vertical farms in the *t-i*-th year;
$x_{t-i}^{5}$ is the growth rate and volume of a product in the *t-i*-th year;
$x_{t-i}^{6}$ is the surface area available for agricultural needs of a system in the *t-i*-th year;
$x_{t-i}^{7}$ is the size of the qualified personnel of a system in the *t-i*-th year;
$x_{t-i}^{8}$ is the investment of a system in the *t-i*-th year.

A system is understood as one or several highly-automated agro-industrial complexes of vertical farming.

With the chosen and substantiated endogenous and exogenous variables a system of equations can be built as a structural model form. In general terms, the listing of the functional dependence of interrelated econometric equations has the following form:

$y_{t}^{1}$ = ƒ($y_{t}^{2},y_{t}^{3},y_{t}^{4},$ $y_{t-j,}^{1}x_{t-i}^{1}$,$x_{t-i}^{2}$, $x_{t-i}^{3},$ $x_{t-i}^{4}$)
$y_{t}^{2}$ = ƒ($y_{t}^{1},y_{t}^{3},y_{t}^{4},$ $y_{t-j}^{2},x_{t-i}^{4},x_{t-i}^{5}$,$x_{t-i}^{6}$)
$y_{t}^{3}$ = ƒ($y_{t}^{1},y_{t}^{2},y_{t}^{4},$ $y_{t-j}^{3}$,$x_{t-i}^{6}$, $x_{t-i}^{5}$)
$y_{t}^{4}$ = ƒ($y_{t}^{1},y_{t}^{2},y_{t}^{3},$ $y_{t-j}^{4},x_{t-i\;}^{7}$,$x_{t-i}^{8}$, $x_{t-i}^{4}$)

Each equation in the model is written in the form of an autoregressive model (ADL). Below is an example for the second equation: $y_{t}^{2}$ =$a_{0}$ +$a_{1}y_{t-1}^{2}+b_{1\;}y_{t}^{1}+b_{3}y_{t}^{3}+b_{4}y_{t}^{4}+a_{4}x_{t-1}^{4}+a_{5}$ $x_{t-1}^{5}+a_{6}x_{t-1}^{6}$.

The methodology for finding the coefficients of interrelated equations is tested on the models of various systems, which are described in [40,41,42].

The methodology for finding the coefficients can be easily applied to the system of one or several highly-automated agro-industrial complexes of vertical farming, located in some territory.

There are many hidden costs connected with the creation of vertical farms and evolvement of vertical farming, which are needed for abstract representation of reality in the mathematical form. The costs are as follows:

(a) Costs of overcoming regulatory barriers.
(b) Understanding all production costs (electricity, fertilizers, wages, etc.).
(c) Costs related to the conformance to various elements of the equipment.
(d) Selecting profitable crops to match the initial capital investment.
(e) Knowing whether to diversify or focus on low hanging fruits (i.e., microgreens).
(f) Costs of entry into the required market.
(g) Assessing the promises of a specific equipment supplier about the return and the time the work on yield will be completed.
(h) Equipment maintenance costs.
(i) Understanding and paying legal service expenses.
(j) Personnel training costs.

Referring to the economic advantages of vertical farms, the mathematical model can be tested using a principle of comparing the economic benefits on the example of individual indicators.

The indicator $x_{t-i}^{5}$ reflects the growth rate and volume of a product. Let us consider growing lettuce. The leaf and root parts of such crops are often tasty and nutritious, and on a farm they are usually gathered 15–20 days earlier than the usual date of harvesting [23]. In vertical farming greenhouse VegataFarm, Tokyo, it takes lettuce just about 40 days to grow instead of the usual 60 days, as a minimum, in a field [43]. More efficient, year round production can quickly adapt to meet the consumers’ demands.

The indicator $x_{t-i}^{4}$ is the cost of land lease. In comparison to the cost of land in the city, it is more profitable to establish innovative farms in suburban regions. The urban fringe is believed to be an innovative space for agriculture. Renting a field for traditional farming is far less expensive than renting spaces in cities. Thanks to the fact that vertical farms radically reduce the length of the logistic chain, food could be stored on the shelfs of shops longer than in case of traditional producers.

The indicator $x_{t-i}^{6}$ is the available farmland that is characterized by erosional processes, which highlights the positive aspect of vertical farming.

The indicator $x_{t-i}^{7}$ is qualified manpower. Less than 40% of agricultural enterprises are staffed with highly-qualified human resources. A lot of foreign rivals on the domestic market have a negative effect on the competitiveness of the national producers.

The indicator $x_{t-i}^{8}$ is the big initial investments. After the decision to invest in a specific vertical farming project has been taken, there is a problem of big initial investments and further expansion of production, which will have a tendency for cost increase. Multi-storey systems of vertical farming can have the same constraints that are obvious for residential high-rise buildings, such as the regulated limits of height or fire safety. The volume of output is not yet considered as a constraining factor in the private case of food products aimed at wealthy customers. The trend for the vertical farming model can increase by 2050 due to climate change and quickly reducing availability of per capita plough land. It is especially important for the poor and deficit grounds in the North. The volume of output and application to planting on wide floor surfaces are the currents constraints on vertical farming [18]. Venture capital can be raised from local investors or investors from abroad, where the interest in clean, green and gourmet (CGG) food is high, where the price of land is in the first place, or where soil and air are polluted. The price of food is not the major constraining factor for wealthy consumers. Indoor farming will, probably, be attractive for financing or co-investing on the part of the government, production sector and universities due to the nature and potential of the projects.

The model of closed environment can be also transferred to remote polar or deserted areas, or even to space exploration, where food production may be needed in spaceships or on other planets [34].

The requirements for the business case of the presented mathematical model include the evaluation of cost-base and profitability through life cycle analysis (LCA) with a traditional farm used as a standard. Moreover, it has to be estimated how many years will be needed to achieve parity with a traditional farm in terms of return on investment (ROI), and study the compromise on the key cost factors affecting the value, including the land, depreciation of plants, market demand and reduction in transportation expenses.

## 5. Conclusions

This study of the structure of nutrition of the population in the northern region of Russia and its health status related to nutrition found out that there is a direct connection with the food security provided to the population.

The authors sociological research in the actual nutrition of the people living in the Arctic region confirms that the actual diet of an Arctic region, such as Yamalo-Nenets Autonomous District is non-rational, unbalanced and characterized by insufficient consumption of fresh vegetables, meat and processed meat products, fish and seafood, milk and dairy products, and unsaturated fats. People eat too much oils and fats, bread and sterols while the quality of food products is relatively stable.

The basis of the food basket of the population of the northern region is bread, milk and fermented milk products, poultry and processed poultry, grocer products and pasta, sausage and smoked products, vegetables grown in a greenhouse. The basis of the food basket is virtually the same for the three upper income quartiles. The lower income quartile demonstrates growing consumption of bread to the disadvantage of all other types of food products. It is highlighted that in comparison with the older generation, the younger consume almost twice as many vegetables and fruit, which are so important in the structure of nutrition.

The health of the people living in the northern regions is directly dependent on the structure and quality of their nutrition. The detailed analysis of the data obtained in the sociological survey shows that the sick people far more infrequently buy fresh fruit and vegetables, berries, dairy products and meat than those who believe they are apparently healthy. An important pattern is observed when different areas are compared by illness incidence. Those who eat a more balanced diet fall ill much less often than the population in other Arctic regions and the infant mortality in these areas is lower.

The cause of the negative result of the chain “structure of nutrition of the population—health status” is in providing food security to the population. The following problems of food security provision to the population are identified. Virtually in all the northern regions of Russia agriculture has negative profitability. Imported foods and food ingredients are mostly used. There are drawbacks of logistics, transportation and storage of food products, caused by the remoteness of settlements and the natural and climatic conditions unfavorable for traditional agriculture. In the rural regions a problem is observed concerning the presence of high quality and fresh food products, primarily dairy products, fruit and vegetables. The analysis shows a low level of self-provision with food products of the people living in the northern territories. Moreover, according to sociological surveys, the problem of the quality of food products annually goes in the top 5 problems of those living in rural regions.

The feasibility study of the stages of life cycle of a highly automated agro-industrial complex of vertical farming in the north shows that one of the possible answers to this challenge is vertical farming, which should solve the problems of organization of food security provided to the population. Highly-automated agriculture will be able to provide the people living in the towns of the Arctic zone with food products. In case of proper conditions, vertical farming has an enormous potential for success. Its introduction will simultaneously help to reduce poverty, increase the safety of food products, context stability and wealth of people providing them with jobs and enriching their food basket with fresh and high-quality food products in the conditions of transport inaccessibility and limitations of agriculturally used areas.

These products are also much cheaper than regular crops because the price does not include transportation or storage costs [44].

In the conclusion, we have to highlight that vertical farms represent a challenge to traditional agriculture, which, in practice, appears due to a multi-layered mechanical system within a high-rise greenhouse. Systems with several stacks have already been operating around the world and are expanding fast. These technologies are sold by several large Japanese firms and supported by the research in a number of universities [36,45,46,47].

## Figures and Tables

**Table 1:** Average assessment scores of the quality and security of food products depending on the self-assessment of the health status.

Self-Assessments of Health Status	Average Assessment of Food Quality
very bad	0.28
rather bad	0.32
rather good	0.34
apparently healthy	0.45
Fisher’s Test	12.38
Sig.	0.000
Pearson’s chi-squared test	0.34
Sig.	0.000

Source: From the authors’ sociological survey.

## Data Availability

The data presented in this study are available on request from the corresponding author.
